# A Surface Plasmon Resonance Biosensor Based on Directly Immobilized Hemoglobin and Myoglobin

**DOI:** 10.3390/s20195572

**Published:** 2020-09-29

**Authors:** Georgi Dyankov, Ekaterina Borisova, Evdokia Belina, Hristo Kisov, Ivan Angelov, Alexander Gisbrecht, Velichka Strijkova, Nikola Malinowski

**Affiliations:** 1 Institute of Optical Materials and Technology, Bulgarian Academy of Sciences, 109 Acad. G. Bonchev Str., 1113 Sofia, Bulgaria; gdyankov@iomt.bas.bg (G.D.); evdokiyabelina@yahoo.de (E.B.); hristokisov@iomt.bas.bg (H.K.); vily@iomt.bas.bg (V.S.); malinowski@iomt.bas.bg (N.M.); 2 Institute of Electronics, Bulgarian Academy of Sciences, 72 Tzarigradsko chaussee Blvd., 1113 Sofia, Bulgaria; ipangelov@orgchm.bas.bg (I.A.); agiz@abv.bg (A.G.); 3 Institute of Organic Chemistry with a Centre of Phytochemistry—Bulgarian Academy of Sciences, 9 Acad. G. Bonchev Str., 1113 Sofia, Bulgaria

**Keywords:** SPR biosensor, direct immobilization, heme proteins, MAPLE deposition

## Abstract

Immobilization of proteins on a surface plasmon resonance (SPR) transducer is a delicate procedure since loss of protein bioactivity can occur upon contact with the untreated metal surface. Solution to the problem is the use of an immobilization matrix having a complex structure. However, this is at the expense of biosensor selectivity and sensitivity. It has been shown that the matrix-assisted pulsed laser evaporation (MAPLE) method has been successfully applied for direct immobilization (without a built-in matrix) of proteins, preserving their bioactivity. So far, MAPLE deposition has not been performed on a gold surface as required for SPR biosensors. In this paper we study the impact of direct immobilization of heme proteins (hemoglobin (Hb) and myoglobin (Mb)) on their bioactivity. For the purpose, Hb and Mb were directly immobilized by MAPLE technique on a SPR transducer. The bioactivity of the ligands immobilized in the above-mentioned way was assessed by SPR registration of the molecular reactions of various Hb/Mb functional groups. By SPR we studied the reaction between the beta chain of the Hb molecule and glucose, which shows the structural integrity of the immobilized Hb. A supplementary study of films deposited by FTIR and AFM was provided. The experimental facts showed that direct immobilization of an intact molecule was achieved.

## 1. Introduction

Heme proteins, such as hemoglobin (Hb) and myoglobin (Mb), have been widely used for sensing non-organic compounds like NO_2_ [1], NO_2_ and H_2_O_2_ [2,3], C_8_H_8_O_3_ [4], NO [5,6], CO [7,8] and HS [9]. Reference [10] has reported a biosensor with Hb as the recognition element (ligand) for sensing organic compounds (glucose). 

To date, Hb and Mb have been immobilized mostly onto electrochemical transducers. Electrochemical biosensors possess high selectivity and biological compatibility but the main drawback is the low voltammetric response because heme groups are deeply buried. One approach to solving the problem is using mediators—small molecular compounds that are electroactive. However, this complicates the electrode process of proteins. Another approach is using a promoter—a small molecular compound which is electroinactive. The effect is orienting proteins toward the electrodes decreasing the distance between the redox center and the electrodes, therefore enhancing the electron transfer. 

A step towards direct electrochemistry of proteins is incorporating them into films that are modified on the electrode surface. In [11] Mb has been embedded in didodecyldimethylammonium bromide (DDAB) films cast on pyrolytic graphite electrodes. The same approach was used to immobilize Hb on a glassy carbon electrode [12]. For Mb immobilization different matrices such as carboxymethyl cellulose (CMC) [13], polytyramine [14], Nafion [15], chitosan [16] were used. These matrices were doped with different conducting materials, such as silver nanoparticles [17], TiC nanoparticles [18] and carbon nanotubes [19]. A highly sensitive detecting platform for a Mb-based biosensor involving graphene oxide-encapsulated molybdenum disulfide nanoparticles has been reported [3]. In [20] a new graphene-like nanomaterial has been used to immobilize hemoglobin (Hb) and fabricate a mediator-free nitrite biosensor. Direct electrochemistry has been provided for hemoglobin (Hb) immobilized on a nanometer-sized zirconium dioxide (ZrO_2_)-modified pyrolytic graphite (PG) electrode [21]. Hb was immobilized on screen-printed carbon electrode pre-electrodeposited with gold nanoparticles (AuNPs) [22].

Hb and Mb have never been used as ligands in surface plasmon resonance (SPR) sensors despite the fact that a synergistic effect of SPR sensor technology and immobilization of Hb/Mb as specific ligands can be expected. This is likely to be due to the ability of proteins to adsorb irreversibly on the untreated metal surface of the SPR chip, often causing loss of 90 or more per cent of their activity [23]. Protein immobilization is a delicate procedure—proteins tend to unfold and denature upon contact with metals and most other artificial substrates. 

However, this statement is valid for conventional methods of immobilization. The matrix-assisted pulsed laser evaporation (MAPLE) method [24] has been successfully applied for deposition of proteins, preserving their bioactivity [25,26]. Blood proteins have been MAPLE-deposited with excellent results [27,28]. It has been proven that laser transfer and deposition preserve protein bioactivity. At the same time, it must be kept in the mind that MAPLE deposition has not been performed on a gold surface. Hence, the effectiveness of MAPLE deposition for SPR chip functionalization requires additional attention.

Conventional immobilization methods require advanced chip architectures since they screen the ligand and analyte from undesirable contact with the underlying metal. However, the complex immobilization matrix raises the question of the SPR sensor selectivity and sensitivity. Moreover, the elaboration of the immobilization matrix is complex, labor-intensive and expensive. Therefore, an immobilization method that avoids the built-in matrix and provides directly immobilized ligands is highly desirable.

In previous publications of ours we reported that MAPLE is a very promising method in this aspect [29,30]. Our preliminary results proved the feasibility of MAPLE for direct Hb/Mb immobilization—we showed that the main functional groups of proteins are present in the deposited film. In [10] we showed the sensing efficiency of directly immobilized Hb, while in [31] we provided supplementary information regarding the immobilization technique. 

In this paper we focus on the impact of direct immobilization on protein bioactivity and the general sensing ability of Hb and Mb, respectively. The successful immobilization of Hb/Mb requires the solution of two problems:

- Preservation of the conformation state. This alters the ability of the molecule to transmit intramolecular messages to its various functional groups, maintaining a maximum affinity for the ligand of interest. For example, oxygen binding depends on the position of the porphyrin in relation to the plane of the heme.
- The effect of dehydration on Hb/Mb activity. The structure of the heme pocket is preserved by the hydrogen bonding of water molecules with the protein surface [32]. Additionally, OH groups bind to the hydration sites of Hb/Mb and prevent conformational changes.

Immobilization by the MAPLE technique can solve these problems at different stages of the immobilization process. The solution of the aforementioned problems can be assessed by registering the bioactivity of the directly immobilized ligands. This is accomplished by SPR registration of the molecular reactions of various Hb/Mb functional groups. By SPR we studied the reaction between the beta chain of the Hb molecule and glucose which shows the structural integrity of the immobilized Hb. In addition to these studies, Fourier transform infrared spectroscopy (FTIR), transmission electron microscope (TEM) and atomic force microscope (AFM) examination of the deposited film were also used. 

## 2. Materials and Methods

A comprehensive review [25,26] of proteins deposited via MAPLE demonstrates the efficiency of this technique. However, to date it has never been used for the deposition of metalloproteins, such as hemoglobin and myoglobin. In MAPLE, metalloproteins are not exposed to laser irradiation, since they are dissolved in a laser power absorbing solvent that is subsequently frozen to form a solid target. In this way, the violent interaction of photons with the biomolecule is diminished since the main fraction of laser energy is absorbed by the solvent. The laser energy is converted into thermal energy that helps vaporize the solvent molecules entraining the protein molecules. The volatile solvent molecules are eliminated by a vacuum system while the protein molecules approach their substrate. The MAPLE set-up is described elsewhere [29,30].

### 2.1. Volatile Solvent

A significant problem is the choice of a volatile solvent in which the proteins are dissolved. Having in mind the reports of results obtained in MAPLE deposition of protein [33,34], we used deionized water in our recent experiments. It is worth mentioning that the quality of deionized water is of primary importance—type I water is required. Liquid water has an absorption band near 1.2 μm, meaning that it is reasonable to use Nd:YAG laser irradiation at 1.064 μm. The absorption of the photon energy by the volatile solvent depends on its absorption spectrum. The frozen target has the absorption spectrum [34] of ice which generally coincides with that of liquid water. However, it is necessary to pay attention to its temperature dependence, since the efficiency of the photothermal process depends on temperature, as discussed below. 

### 2.2. Target

We have used a frozen target consisting of 5–7% Hb/Mb solution in deionized water. To study the impact of direct immobilization on protein conformation, in the first place we considered the MAPLE parameters which determine the status of proteins remaining in the target holder after deposition, without exposure to laser irradiation. We have shown [31] that the parameters (called “optimal”), which do not change the chemical structure of the target substance are as follows: target temperature −40 °C and downtime in the vacuum camera 45 min (at pressure less than 10^−3^ Torr). Also, the cooling rate is very important, as discussed below. The conclusions are based on the analysis of FTIR spectrum—the spectrum of the starting substance (lyophilized Hb) was chosen for comparison.

### 2.3. Deposition of Bioactive Ligands

Once optimal parameters have been established, we proceeded with studying the impact of *MAPLE* processing parameters that define the structural composition and bioactivity of the deposited molecules. The most important parameters are pulse energy, pulse repetition rate and pulse duration. Since the target absorption increases with decreasing the temperature [35], higher efficiency of the photothermal process occurred at lower temperatures and proteins were preserved from distortion. For the Hb solution target with a temperature of −30 °C, the optimal pulse energy was 44 mJ, while at −40 °C it was 35 mJ, with the other parameters kept constant: the optimal pulse duration was 10 ns, the optimal repetition rate was 10 Hz, fluence was 160 mJ/cm^2^, with pulses varying between 18 k and 47 k. For the Mb solution the parameters were different, as discussed in Section 3.

**Figure 1:**
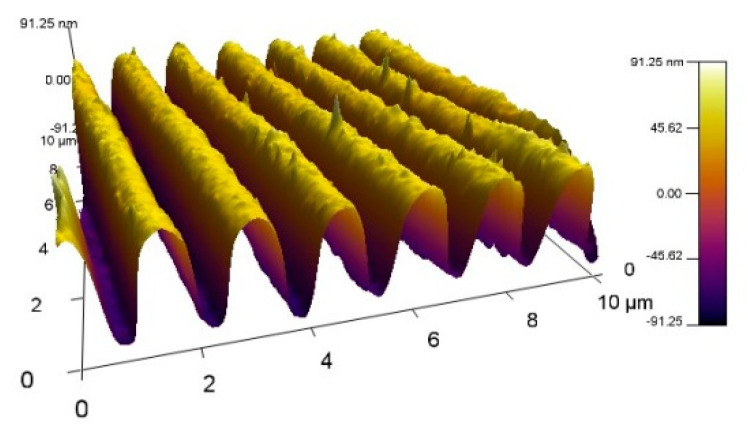
AFM scan of a gilded diffraction grating covered with a 120 nm thick myoglobin (Mb) film.

The bioactivity of MAPLE-deposited proteins is the main criterion for the efficiency of immobilization. Immobilized molecule bioactivity was assessed by SPR registration of the binding ability of the various functional groups of metalloprotein molecules with carbon monoxide (CO), carbon dioxide (CO2), nitric oxide (NO) and oxygen (O2), as reported in [29,30]. For this purpose, gilded diffraction grating was used as a facing substrate over which a film of Hb/Mb molecules was deposited. Polycarbonate substrate for a CD-R disk covered with a gold film, 80–110 nm in thickness, was used as diffraction grating. The grating was immersed in isopropyl alcohol and cleaned ultrasonically before the MAPLE deposition process. An AFM scan of the grating with a directly immobilized Mb film is shown in Figure 1.

Another criterion is the adhesion quality of the film deposited on the gilded grating. The quality is evaluated by the solubility of the film when sonicated in water for 30 s. If this procedure does not impact SPR, adhesion is considered to be good. Achieving good adhesion involves balancing laser energy and target-substrate distance. Simultaneously, laser energy impacts the bioactivity of deposited films. Consequently, the optimization procedure is complex, since it requires a trade-off between a number of parameters, as discussed in Section 3.

### 2.4. Chemicals 

Hemoglobin (from bovine blood, H2500) and myoglobin (from equine skeletal muscle, M0630) were purchased from Sigma-Aldrich (Sofia, Bulgaria, FOT Ltd.-representative). The pure gases and gas mixtures (CO, CO_2_, NO, O_2_) required were purchased from Messer Bulgaria (Sofia, Bulgaria).

## 3. Results and Discussion

### 3.1. Impact of Freezing Rate

Not only the target temperature, as mentioned above, but also the freezing rate influences the laser energy required for successful film deposition. We found that shock freezing required lower pulse energy to preserve bioactivity. A possible explanation is that the rapid process makes the target substance more inhomogeneous in terms of the energy state of protein molecules. Then, the energy absorbed by the molecules dissipates in a plurality of molecular vibrational states that make the conversion of photon energy into thermal energy more effective. This effect is more pronounced for the Hb solution as compared to the Mb one. Hb molecules, being more complex and larger, possess many conformation states that can be affected by energy dissipation, as a result of which the molecules can be effectively heated. 

### 3.2. Protein Molecules Take Part in the Photothermal Process: The Impact

#### 3.2.1. Activity of the Heme Group

The different conditions for Hb and Mb immobilization that we identified indicate that the photothermal process is not limited only to the volatile solvent molecules—protein molecules are also involved. The induced conformational states related to the heme group can be documented by registration of CO or O_2_ binding to the heme. Since heme bioactivity depends on the position of porphyrin, the absence of binding reactions indicates an undesirable conformational state. Laser power is not the only factor that can affect the conformational state. Dehydration, which is inherent in a vacuum, detaches water molecules from the heme pocket, which results in formation of a new conformational state [32]. Indeed, we observed low or even no bioactivity of the heme group (i.e., no CO binding reaction was registered), while activity of other functional groups was observed. 

#### 3.2.2. Role of Molecular Weight

**Figure 2:**
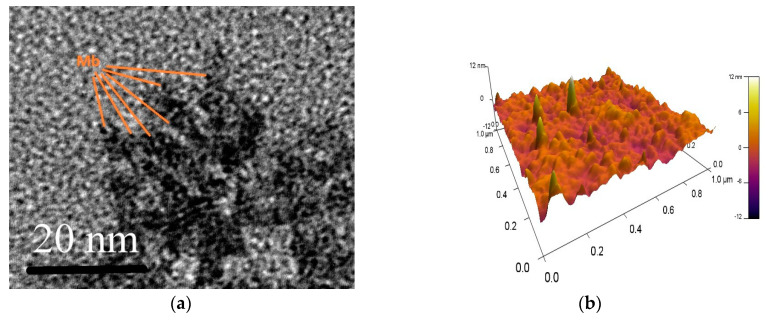
(**a**) TEM images of a Mb cluster deposited under conditions of optimal Hb immobilization; (**b**) AFM image of the Mb film deposited under corrected conditions.

Experimentally, it is more convenient to apply shock freezing to the target. In this regime we observed distinct difference in the laser power required for successful Hb and Mb deposition. Figure 2a shows a TEM image of a Mb structure deposited at values of a set of parameters which provide successful Hb immobilization. More precisely, it shows one of many similar clusters that were observed–no continuous film was deposited. The round particles (marked), having an average size of about 5 nm, were identified as Mb molecules. The MAPLE-deposited Hb film had no cluster structure, which can be explained by the aforementioned differences in energy dissipation associated with the photothermal process. Although the MB layer with a cluster structure showed bioactivity, the aim is to deposit a dense film that covers the gilded lattice. This avoids possible non-specific adsorption of the ligand on the metal surface.

After the pulse energy was corrected with a coefficient compatible to the ratio of Hb/Mb molecular masses, Mb film bioactivity was improved and continuous nanostructured Mb film was formed. The AFM micrograph (Figure 2b) illustrates the structure of the Mb film.

### 3.3. Characterization of Immobilized Proteins

Laser transfer and direct immobilization of protein molecules are characterized by FTIR and SPR-detected binding reactions. As mentioned above, we managed to establish MAPLE optimal parameters under which there is no impact on the chemical structure of the protein molecules left in the target holder after the immobilization process, as well as no exposure to laser irradiation [31]. It follows that the bioactivity of molecules immobilized at optimal parameters is a consequence of the interaction between protein molecules and laser irradiation. The impact of the interaction was evaluated by studying the chemical and structural properties of the deposited films.

#### 3.3.1. FTIR Spectra 

**Figure 3:**
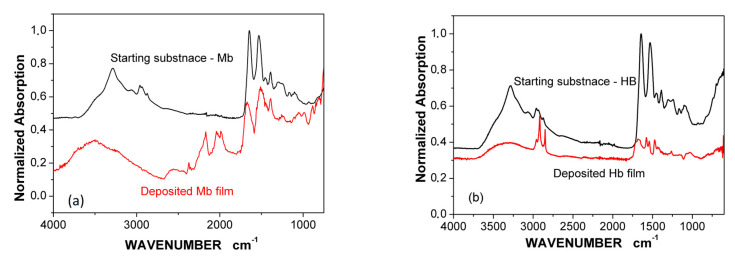
FTIR spectra of the starting substance and: (**a**) the deposited Mb film; (**b**) the deposited Hb film.

Figure 3a compares the FTIR spectra of the starting substance (lyophilized Mb) with the spectra of the Mb film deposited (on a Si substrate). The Si substrate is adjacent to the gilded diffraction grating and one can assume that the deposited films on both surfaces are identical. Obviously, the main characteristic bands are expressed; however, some are missing. These are our best results achieved with MAPLE immobilization. Similar results have been reported for Hb film in [31], shown in Figure 3b for comparison. It is relevant to note that we found more favorable conditions for immobilization of Mb molecules than for Hb molecules. The spectra of Mb films observed were closer to the FTIR spectra of the starting substance than those of the Hb films. The conditions established for successful deposition of Hb and Mb differed. According to FTIR spectrum, deposited films presented a chemical structure close to the starting materials. As expected, the laser fluence plays a critical role not only in film morphology (Figure 2a) but in conserving the chemical structure. The optimal fluence is mentioned in Section 2.3.

#### 3.3.2. SPR-Detected Binding Reactions

**Figure 4:**
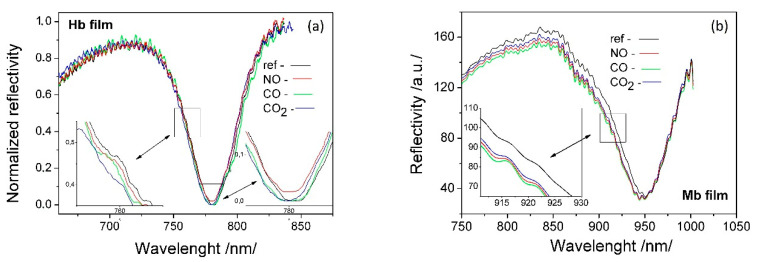
SPR resonance shift resulting from the binding of NO, CO and CO_2_ to corresponding functional groups of: (**a**) Hb molecules; (**b**) Mb molecules.

It is interesting to evaluate to what extent the observed differences in the FTIR spectra (Figure 3) are related to the bioactivity of the deposited Hb/Mb films. For this purpose, the bonding of carbon monoxide (CO), carbon dioxide (CO_2_), and nitric oxide (NO) to the corresponding Hb/Mb functional groups were studied by SPR. Figure 4 shows the spectral shift of the plasmon resonance resulting from the binding reaction between NO (700 ppb), CO (500 ppm) and CO_2_ (1000 ppm) and Hb/Mb molecules. The details regarding SPR set-up and registration were the same as those previously reported by us [29,30].

The deposition of the Mb film on the SPR transducer and the film on the Si substrate, the FTIR spectrum of which is shown in Figure 3a, occurred in one and the same MAPLE process. CO binding was used to illustrate the bioactivity of the directly deposited molecules since the heme group is most sensitive to conformation changes. The bioactivity of the amine and thiol groups was studied via the SPR spectral shifts observed, all shown in Figure 4b. A registered spectral shift of about 5–8 nm indicates the activity of the deposited ligands and is an integral criterion for the efficiency of the direct immobilization. 

**Figure 5:**
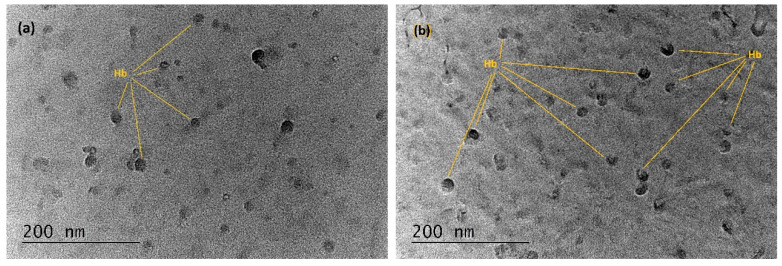
TEM images of (**a**) the Hb film deposited by spin coating; (**b**) the MAPLE-deposited Hb film.

The structure of the deposited films was studied by TEM. Figure 5a,b show images of spin-coated and MAPLE-deposited films, respectively. In both cases, the concentration of the Hb solutions was the same, and the films had equal thickness. The round particles observed (marked) of an average size of about 20 nm were identified as Hb molecules. Image analysis of TEMs followed by statistical analysis indicated that the density of the deposited Hb molecules of the spin-coated film was higher than that of the MAPLE-deposited film. It can be assumed that the missing molecules in the MAPLE film have been destroyed by the laser transfer.

### 3.4. Effectiveness of MAPLE Deposition 

**Figure 6:**
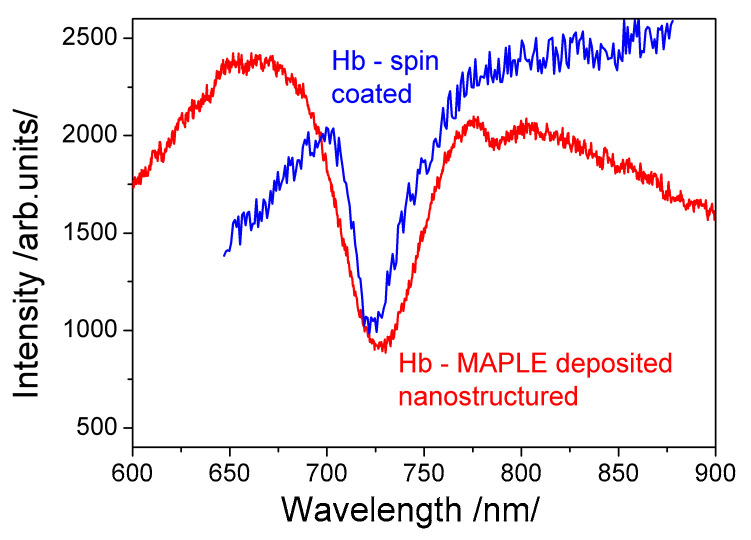
SPR excitation comparing the effectiveness of both immobilization techniques: MAPLE (red curve) and spin coating (blue curve).

This statement is supported by SPR observed in films deposited on gilded grating. Figure 6 shows that SPR excited in a MAPLE-deposited film has a spectral width two times wider than that of the spin-coated film. Since the resonance width is a measure of the dissipation losses, they are obviously higher in the MAPLE-deposited film due, probably, to the fragmented Hb molecules. Statistical analysis for a hypothesis about difference between two population means was conducted. 35 random samples with normally distributed molecules were analyzed and the normalized deviation is 1.96. Hence, the population of molecules in spin-coated film is higher that MAPLE population with confidence level of 96%. Can be assumed that the difference is due to destroyed molecules caused by the laser transfer.

Besides, the measured resonance spectral shift originating from binding reactions in the MAPLE-deposited film was more than twice smaller than the one observed in the spin-coated film. This supported the statement that the number of bioactive molecules in a MAPLE-deposited film is lower. As a consequence, the SPR shift is not well pronounced. 

In [29,30] we showed that Hb and Mb directly immobilized on gilded diffraction grating maintain their bioactivity. As mentioned above, the vitality of heme, amine and thiol groups was explored by SPR registration of the binding reaction with CO, CO_2_, and NO, respectively. However, these experimental facts cannot prove the integrity of the molecules deposited—the bioactivity observed could be a consequence of fragmented Hb/Mb molecules that have the above-mentioned functional groups. 

### 3.5. What Molecules are Immobilized—Fragmented or Intact? 

**Figure 7:**
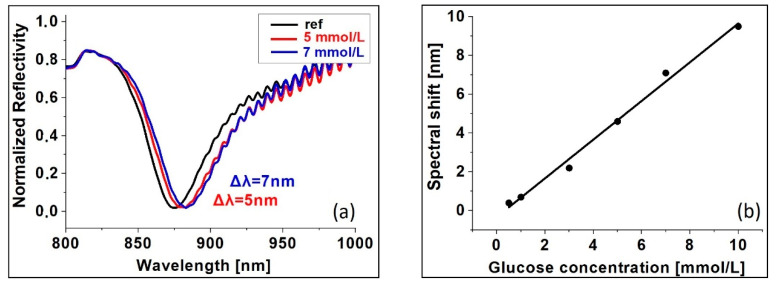
SPR registration of Hb-glucose binding; (**a**) SPR at different glucose concentrations; (**b**) SPR shift vs glucose concentration.

For the purposes of providing convincing experimental evidence of an intact molecule immobilization, we studied the interaction between Hb and glucose. Glucose covalently binds to hemoglobin through the N-end of the Hb molecule beta chain [36] and forms glycated hemoglobin. To study the binding reaction, Hb was directly immobilized on gilded diffraction grating. Figure 7a shows SPR response vs. glucose concentration. The black curve is the resonance of the so-fabricated SPR chip, while the color curves show the resonances at different glucose concentration. The wide spectral width of the resonance limited the sensitivity and accuracy of measurement. Figure 7b demonstrates achieved low detection limit–it is 0.5 mmol/L glucose concentration. The SPR response is evidence of the bioactivity of the Hb molecule beta chain. Since the bioactivity of other functional groups was also experimentally proved, one can conclude that intact direct immobilization was observed. 

A reasonable question is whether directly immobilized ligands perform better in terms of sensitivity. In our experiments we achieved 28 Da (for CO). Compared with SPR sensors based on conventionally immobilized ligands that possess low response induced by binding of smaller analytes with a molecular mass less than 1000 Da, our result is a convincing proof of the higher performance of directly immobilized ligands. Also, the low detection limit was established as follows: 30 ppm for CO and 70 ppm for NO. The comparison with previously reported results is not possible since the sensing is based on different methods and incomparable units of measurement are used [6,7]. 

The performance of directly immobilized Hb is confirmed by the achieved very sensitive glucose detection with low detection limit 0.1 mmol/L [10]. We cannot compare our results with other reports, since we were first to use hemoglobin as a ligand for glucose detection.

## 4. Conclusions

Our attention was focused of the impact of direct immobilization on Hb and Mb bioactivity. FTIR spectra, TEM and AFM techniques were used to characterize it. Although FTIR spectra indicated that deposited films did not entirely preserve the proteins’ chemical structure, they possessed suitable bioactivity for sensor applications. So far, this bioactivity is smaller than the activity of films immobilized by spin-coating, hence the immobilization process parameters need to be further improved so that ligand molecule fragmentation could be avoided. 

By studying the specific binding reaction via SPR, the bioactivity of directly immobilized ligands was confirmed. To the best of our knowledge, this was the first time Hb was used to detect glucose. Glucose binding to Hb suggested that intact molecule immobilization was achieved. 

Since the first years of development of the SPR technology, it has been generally accepted that high sensitivity and selectivity of detection cannot be achieved through direct immobilization. This is likely due to the chemical/biological immobilization methods that have been used. We showed that the MAPLE technique provides unique opportunities for direct immobilization not only due to its ability to preserve the bioactivity of the immobilized molecules but also because it provides good adhesion between the deposited molecules and the substrate. The feasibility of directly immobilized Hb and Mb was demonstrated by achieving ultra-sensitive detection of glucose and the SPR sensing of light molecules as CO and NO.
